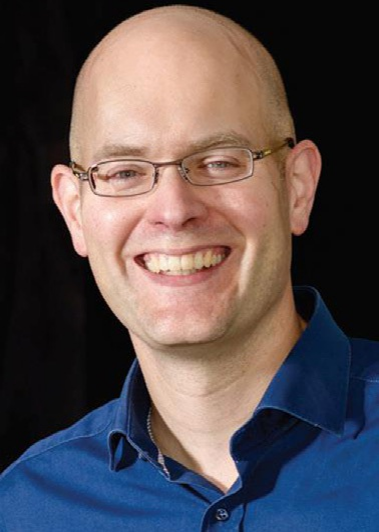# Technology Trends of Catalysts in Hydrogenation Reactions: A Patent Landscape Analysis

**DOI:** 10.1002/adsc.201901292

**Published:** 2020-02-12

**Authors:** Marius A. Stoffels, Felix J. R. Klauck, Thomas Hamadi, Frank Glorius, Jens Leker

**Affiliations:** ^1^ Institut für Betriebswirtschaftliches Management im Fachbereich Chemie und Pharmazie Westfälische Wilhelms-Universität Münster Leonardo-Campus 1 48149 Münster Germany; ^2^ Organisch-Chemisches Institut Westfälische Wilhelms-Universität Münster Corrensstraße 40 48149 Münster Germany; ^3^ Institut für physikalische Chemie Universität zu Köln Luxemburger Str. 116 50939 Köln Germany

**Keywords:** catalysis, hydrogenation, patent landscape, patents, technology

## Abstract

The purpose of this review is to present an overview of the patent landscape for catalysts used in hydrogenation reactions. Based on patent data extracted from PatBase®, we use predefined patent classifications as well as a keyword‐based search for our analyses. The results indicate that the number of patent families that protect heterogeneous catalysts grows twice as fast as that for their homogeneous counterparts. Furthermore, the data show a shift towards abundant and non‐toxic elements in heterogeneous catalysis, while the noble metals continue to dominate the patent landscape of homogeneous catalysis. A subsequent geographical analysis reveals that the high growth rates in heterogeneous catalysis, especially for nickel and iron, are driven by China. Conversely, patenting activities with regard to homogeneous catalysts mainly take place in the USA, the EU, and Japan. The subsequent keyword‐based search illustrates the continuous industrial relevance of enantioselective hydrogenation and transfer hydrogenation, as well as the rapidly increasing body of patents in hydrodeoxygenation. Setting these finding into context, we present and apply two concepts that are commonly used in patent analyses, namely the technology life cycle and the S‐curve. We conclude that hydrogenation catalysis has not reached its peak economic relevance yet and will continue to spark valuable patents and innovations in the future.

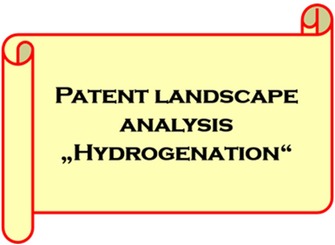

## 1 Introduction

Catalysis is a key technology in chemicals and pharmaceuticals. It is estimated that for over 90% of all chemical products, the production process involves at least one catalytic fabrication step,^1^ and not seldom, this is a hydrogenation reaction.

First defined by Berzelius in 1835, later refined by Ostwald, a catalyst is a chemical entity that speeds up a chemical reaction without being itself changed in the course of the process.^2^, ^3^, ^4^ It does not influence the thermodynamics of the overall reaction but permits a previously inaccessible reaction path. This can lead to a dramatic increase in the reaction rate and enables chemical transformations that are otherwise not feasible. The advances in the theory and application of the concept of catalysis in the 19^th^ century culminated in the Nobel prize for Wilhelm Ostwald in 1909.^4^

On a general level, catalysts are characterized as homogeneous or heterogeneous, depending on whether they exist is the same phase or in a different phase than the reactants. A homogeneous catalyst is in most cases a soluble chemical entity that speeds up the reaction of reactants in the same, typically liquid, phase. Prominent examples include molecular transition metal complexes such as Wilkinson's catalyst, Noyori‐type catalysts, Crabtree's catalyst, and Schrock–Osborn's catalyst, among others. Heterogeneous catalysts are in most cases solids, or mixtures of different solid components that are not in the same phase as the reactants. In this domain, Raney nickel and Pd/C are well established. Typically, the reactants are dissolved in a liquid or gaseous phase and are brought into contact with the solid catalyst to effect an increase in reaction rate.^5^ In the chemical industry, the vast majority of catalytic processes involve heterogeneous catalysts due to their advantage of being easily removable from the reaction mixture by physical solid‐liquid separation techniques.^6^ Heterogeneous catalysts can be distinguished by the fraction of catalytically active material the solid contains. Full catalysts consist solely of the catalytically active material, whereas supported catalysts also contain other species, which may have a catalytically relevant function but can also merely serve as a carrier. These two realms of catalysis (homogeneous *vs*. heterogeneous) require distinct technologies for the preparation, the analysis and the application of catalytic bodies.

In this work, we analyze the patent activity in the field of catalytic hydrogenation. To the best of the authors’ knowledge, this work constitutes the first analysis of the hydrogenation patent landscape to date. Patent reviews have been established as a tool for assessing the *status quo* of scientific research areas that have accomplished widespread industrial application.^7^ Prior patent reviews have been conducted for industrially relevant research fields such as biodegradable polymers,^8^ lithium ion batteries,^9^ and organic photovoltaic cells,^10^ among others. While this type of publication strives to present an overview over technological developments in the respective field, delivering a detailed technology assessment is often out of scope. Therefore, the aim of this review is to present a comprehensive overview of catalyst classes and their evolution for the field of catalytic hydrogenation.

The hydrogenation reaction, i.e., the addition of dihydrogen across an unsaturated moiety or a functional group in a molecule, is frequently applied in the chemical industry. Often, the reaction is only feasible in the presence of a catalyst (stoichiometric reductions by diimide species are known,^11^ but are of low economic significance). Even though frustrated Lewis‐pair species have also been evaluated as hydrogenation catalysts,^12^ the importance of transition metal catalysts outweighs these recent developments.

The paper is structured as follows: As hydrogenation can be effected by both homogeneous and heterogeneous catalysts, we start by presenting examples for these reaction types, followed by a brief introduction into the use of patent analysis. The research design will then be explained and the technological landscape among catalysts in hydrogenation will be presented. Finally, we complement the classification‐based analysis with a keyword‐based approach.

## 2 Theory

### 2.1 Introduction to Hydrogenation

**Scheme 1:**
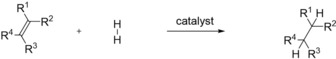
Exemplary, schematic representation of olefin hydrogenation. Other functional groups are also included in the analyses.

Hydrogenation describes a chemical reaction that involves the addition of dihydrogen (H_2_) to an unsaturated moiety (see Scheme 1). The product of this type of reaction is a molecule that bears two or more additional hydrogen atoms in its molecular architecture.

The development of catalytic hydrogenation reactions is an outstanding example for how chemical research influences industry practices and, subsequently, has been honored with two Nobel prizes (i.e., Sabatier 1912, Knowles and Noyori in 2001).^13^, ^14^, ^15^ Today, also represented by the large number of patents filed, catalytic hydrogenation reactions are used extensively throughout the chemical industry. The examples presented in the following underline the essential role of hydrogenation reactions for the production of commodity and specialty chemicals.

#### 2.1.1 Benzene Hydrogenation to Produce Cyclohexane

**Scheme 2:**
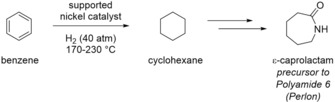
Industrial process of catalytic benzene hydrogenation in the value chain of Nylon production.

The catalytic heterogeneous hydrogenation of benzene to cyclohexane is an example of a large‐scale hydrogenation reaction in the production of commodity chemicals. Cyclohexane is a cyclic hydrocarbon that is also an intermediate for the production of *ϵ*‐caprolactam. This compound is required for the production of Nylon fibers and resins (see Scheme 2).^6^ Typical reaction conditions involve the application of a supported (heterogeneous) nickel catalyst at temperatures between 170 and 230 °C at a hydrogen pressure of 40 atm.^16^

#### 2.1.2 Production of l‐DOPA by Enantioselective Catalytic Hydrogenation

**Figure 1:**
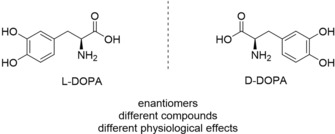
l‐DOPA and d‐DOPA, enantiomers showing distinct physiological effects to humans.

An instructive example for the industrial application of catalytic homogeneous hydrogenation is given by the production of l‐DOPA, a drug used to treat Alzheimer's disease. The drug molecule bears a stereogenic center so that image and mirror image of the compound are not superimposable (Figure 1). The two species are referred to as enantiomers and have different effects on the human organism, even though most physical properties are the same. Since only the l‐enantiomer exhibits the desirable activity, a method for the selective production of this enantiomer was required (Figure 1).^17^

**Scheme 3:**
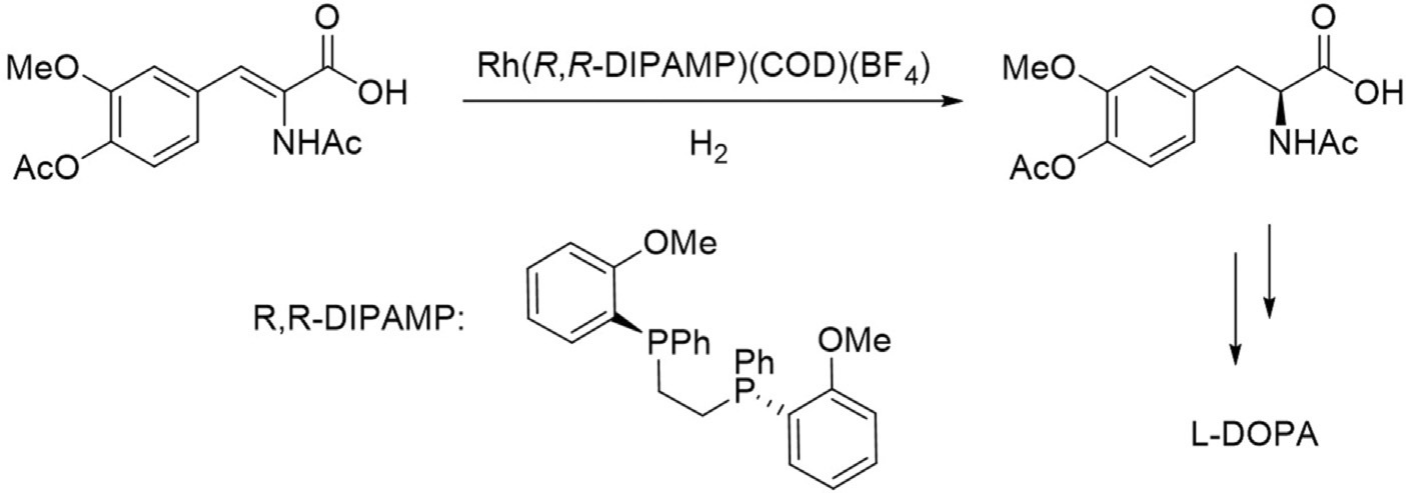
Process for the enantioselective production of l‐DOPA.

In the 1970s, Monsanto patented a process for the enantioselective production of l‐DOPA. The industrial process gives access to the l‐enantiomer selectively by aid of a homogeneous rhodium‐catalyzed hydrogenation of the precursor acetamidocinnamic acid derivative (Scheme 3).^18^

### 2.2 Introduction to Patents and Patent Analysis

Patents are a major force of competitive advantage in knowledge‐intensive industries such as chemicals and pharmaceuticals. They constitute a form of intellectual property and grant the patent's owner the right to exclude others from exploiting an invention in one or more geographical areas for a distinct period of time. Furthermore, patents are an important indicator of technological evolution.^19^ For this reason, it is worthwhile to analyze the patent landscapes of technology areas.

In order to qualify for patent protection, an invention must meet the following three core criteria besides its general patentability: novelty (not “state‐of‐the‐art”), usefulness (susceptible of industrial application), and non‐obviousness (involvement of an “inventive step”).^20^ Patent protection for a specific geographical region is granted by the respective national or regional patent office, for example, the United States Patent and Trademark Office (USPTO) or the European Patent Office (EPO). The date of the initial patent application is called the *priority date,* also referred to as the *priority*. After this initial application, further national, regional and international filings with referral to the priority can be made. Patent applications that refer to the same priority are called *patent families*. In most countries, the invention is protected for 20 years and the patent is publicly disclosed after 18 months.

Patents are a relatively reliable source of technical information, since they are typically subjected to thorough legal examinations. Although it is known that single patents may contain erroneous information, and different patenting strategies exist, the body of the patent itself – as will be used for our analyses – can be regarded as one of the most reliable sources of publicly available technical information on hydrogenation catalysts.^19^ Consequently, the analysis of patents is a well‐established approach for assessing technological change and forecasting the trajectories of emerging technologies.^21^, ^22^, ^23^, ^24^ A suitable indicator for the development of patent activity is the number of patent applications over time.^19^, ^20^

**Figure 2:**
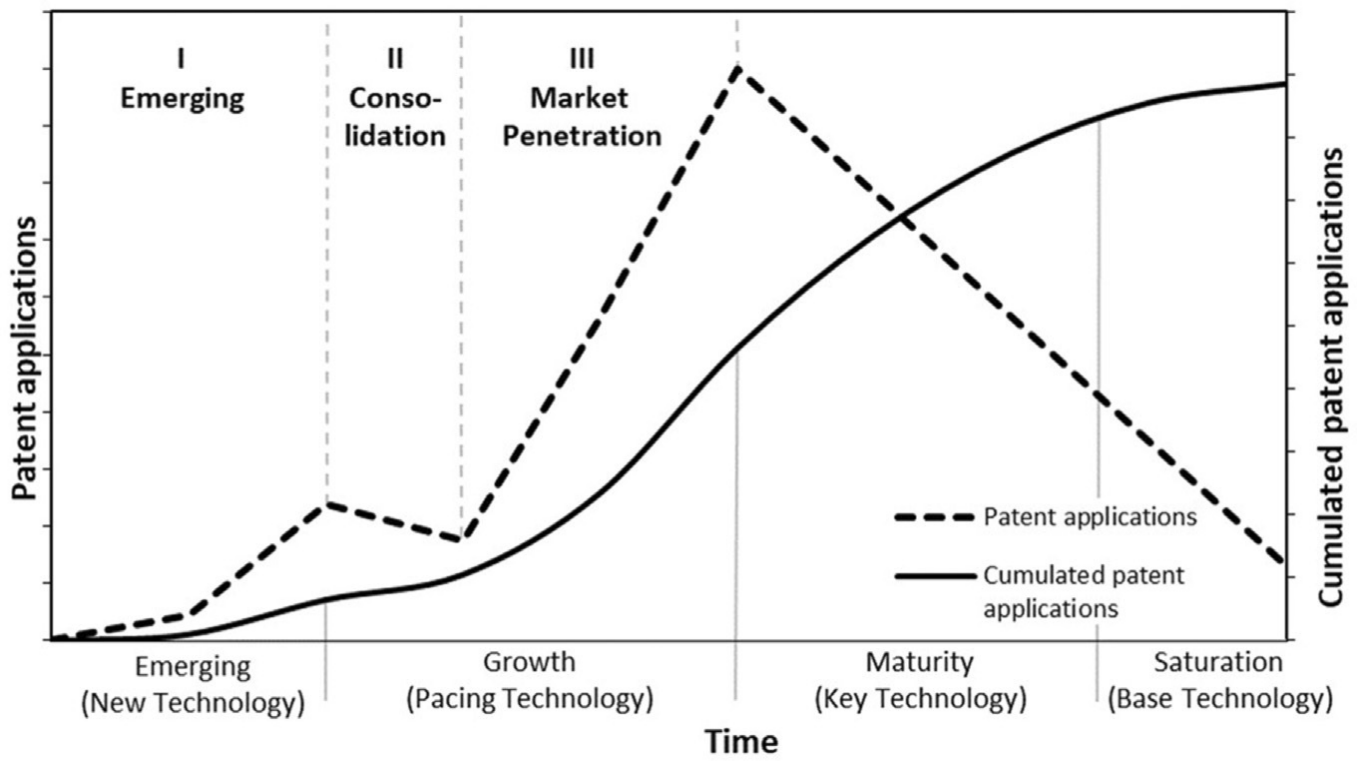
Theoretical development of patenting activity over time and S‐curve concept of a technological life cycle.^19^

In this context, the technology life cycle concept and the S‐curve concept have been established as two theoretical lenses. According to the technology life cycle – as indicated by the dotted line in Figure 2 – the evolution of patent activity for any technology goes through three stages.^19^ These include first, an “emerging” phase with accelerating patenting activities, second, a subsequent consolidation period coined by a slower increase or even a decline in patents, and third, a market penetration phase in which activities reach their peak before they slowly decline again. Furthermore, an S‐shaped (sigmoidal) relationship of technological performance (or cumulative R&D expenditures) over time has been reported (see Figure 2).^9^, ^19^ According to this concept, the technological performance equally runs through different stages. In the emerging stage, technological performance is low and most effort is devoted to basic research. The following growth stage is characterized by an accelerated growth rate (pacing technology), while in the maturity stage performance improvements flatten out (key technology). In the saturation stage, the technological performance starts to stagnate and further technological performance improvements require high R&D efforts (base technology). Since technological performance improvements are often costly to unlock in the maturity and saturation stage, further R&D investments might only pay off for firms with sufficient production scales.^19^

While the number of patent applications is an indicator for the industrial interest in a particular technological field,^25^ it does not give any information about the economic quality of the patents. For the evaluation of the patent quality, we use the share of triadic patents as an indicator, which is a common approach for assessing the economic value of a specific technological field.^8^, ^25^ Triadic patents are patents that are registered at the patent offices in the USA (USPTO), in Europe (EPO), and in Japan (JPO).^26^ The logic is that if a patent has been filed in these three strong economies, it must be of high industrial relevance, which is also referred to as the patent's quality.

## 3 Research Design

For this analysis, the online patent‐database PatBase® was used. This database provides access to patent documents from over 100 issuing authorities worldwide and contains more than 47 million patent families. If patents contain common priorities with other patents, PatBase® groups them into families. These extended families are used by the European Patent Office (EPO) and have the advantage of de‐duplicated and pre‐grouped results.

In order to create a comprehensive and exhaustive dataset for subsequent analysis, we followed the procedures described in prior literature.^9^ Specifically, we used the International Patent Classification (IPC) and Cooperative Patent Classification (CPC) in combination with a search term in the title, abstract, and claims fields. Based on their content, patents are equipped with at least one but usually several of these IPC/CPC classifications to increase their retrievability and group them into technologically related categories. The CPC system is the result of a joint harmonization effort of the European and US patent offices. It was recently introduced and allows a more detailed search among technologies compared to the IPC system.^27^ Classification codes are based on textual contents and provide more objectivity in patent searches. Classification systems are, however, limited in their depth of detail and degree of differentiation.^9^, ^28^ Therefore, they are able to provide a general overview of the developments in the field and can be expanded by more specific analyses or distinct research areas.

Acknowledging these limitations, we used a combined classification‐ and keyword‐based approach of IPC and CPC codes and precise keywords.^9^ Boolean operators as well as truncation were included in this patent search. The keyword search was either based on the full text (FT) or on the title, abstract and claims (TAC) of each patent document.

To further distinguish between heterogeneous and homogeneous hydrogenation, we used several IPC and CPC codes, which allow a distinction of the catalyst nature and phase of the catalytic body. Furthermore, in the case of homogeneous hydrogenation, we also excluded all supported reactions and all patents which were found to be in the field of heterogeneous hydrogenation. Hence, we used the clearly defined IPC and CPC codes to differentiate between heterogeneous and homogeneous catalysts. It might occur that some patents describing a homogeneous complex for the reaction of interest actually involve a heterogeneous active species in the reaction and *vice versa*. However, by checking samples of the respective results manually, we conclude that this instance does not threaten the validity of our findings.

## 4 Analysis and Results

In the following analyses, we categorize hydrogenation catalysts according to their IPC and CPC codes and discuss developments among the different types of catalysts employed. First, we show the overall patenting rate in catalytic hydrogenation, and in heterogeneous and homogeneous hydrogenation specifically. Then, we present major developments in industrial R&D among the manufacture of catalysts, the catalysts themselves, and catalyst investigation and further manipulation.

### 4.1 Developments in the Field of Catalytic Hydrogenation

**Figure 3:**
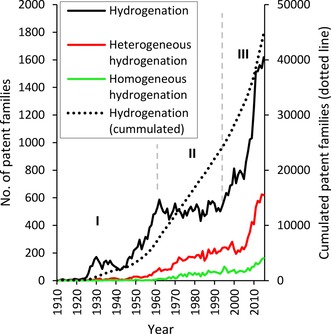
Annual patent activities in the field of catalyzed hydrogenation, heterogeneous hydrogenation, and homogeneous hydrogenation reactions. Due to the strict parameters applied for the homogeneous/heterogeneous classification, the two do not add up to the overall curve.

Starting with a general overview, Figure 3 presents annual patent activities in the field of catalytic hydrogenation. Probably one of the first and still one of the most important processes that involve catalytic hydrogenation is the Haber–Bosch process introduced into chemical production in 1913. Ammonia is produced by the catalytic addition of hydrogen to nitrogen from air over a heterogeneous catalyst. Without this process, the tremendous need of a growing population for synthetic fertilizers could not have been satisfied.^29^ Figure 3 shows a growth in patent family applications for hydrogenation from 1925 onwards. [NB: In this further analysis, patent family applications are termed as patent families.] In terms of the technology life cycle, this is the emerging phase (**I**) during which hydrogenation catalysts started to become utilized in the chemical industry. In this emerging phase, which lasted until around 1960, a growth in patent activity can be observed and more companies started to enter this technological field. From then on the patent activity stayed mostly constant until 1993, indicative of a consolidation phase (**II**). Here, R&D expenditures are reduced and efforts refocused based on the experience in the application of catalytic hydrogenation. Beginning in the mid 1990s, a rapid growth in patent activity is observed, indicating the transition into the market penetration phase (**III**). According to the S‐curve concept, catalytic hydrogenation technologies can be considered to be in the end of the growth stage, where the competitive impact is high and the integration into process technologies is ongoing.^19^

In this analysis, we focus on the nature of the precatalysts, since they are clearly distinguishable as heterogeneous or homogeneous. As shown in Figure 3, heterogeneous hydrogenation accounts for a higher patent activity compared to homogeneous hydrogenation. Furthermore, looking at the development of the annual number of patent families, the curve for heterogeneous hydrogenation follows the same shape as the curve representing catalytic hydrogenation. This result is not surprising, as the heterogeneous nature of solid catalytic bodies inherently possesses advantages over homogeneous catalysts. Most notably, they are easily separable from the reaction mixture and therefore reusable, they enable a continuous processes design with a stationary catalyst, and eventually show superior economic properties. The overall hydrogenation curve also includes patents that are not in one of these clearly defining classes, so the homogeneous and heterogeneous curves do not add up to the overall cumulated number of patents in the field of catalytic hydrogenation.

**Figure 4:**
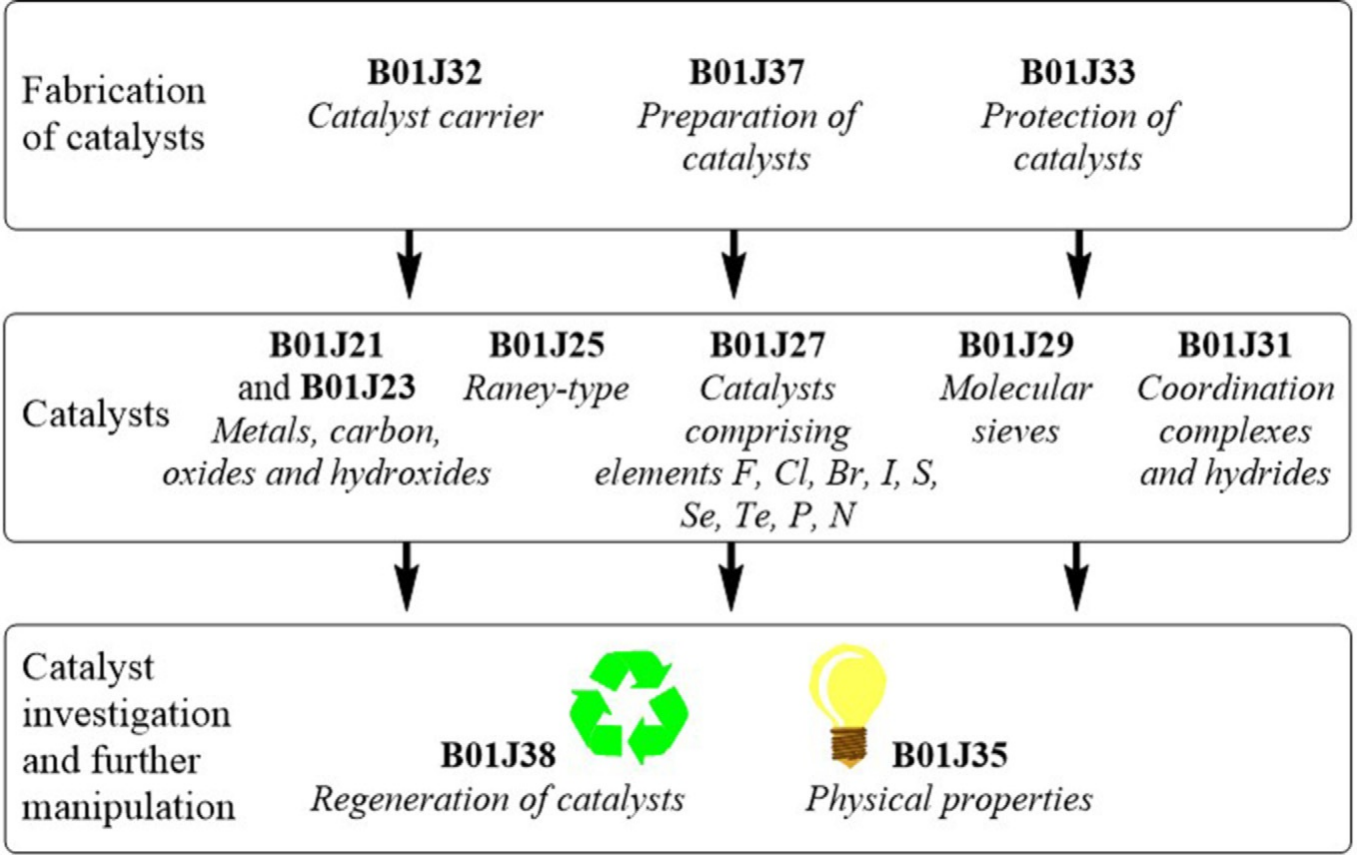
Analyzed research fields among catalysts in the field of hydrogenation and their corresponding International Patent Classifications.

To give a more detailed analysis of the patent landscape in catalytic hydrogenation, we differentiate the field of catalytic hydrogenation according to three different research fields: (i) fabrication of catalysts, (ii) catalysts, and (iii) catalyst investigation and further manipulation. In the next section, the fabrication of catalysts, we take a closer look at catalyst carriers and the preparation and protection of catalysts. Afterwards, we discuss developments among different catalysts materials, such as Raney‐type catalysts, metal oxides, and coordination complexes. Here, we make a detailed analysis of the different metals used in oxides and coordination complexes and their economic and technological quality. We then present an overview of the patent landscapes for the catalysts’ physical properties and the regeneration of catalysts. All of these research areas are classified according to IPC and CPC codes, which allow a distinction of these fields. Figure 4 shows an overview of the three different research areas among catalysts and the underlying International Patent Classifications.

### 4.2 Fabrication of Catalysts

#### 4.2.1 Preparation of Catalysts

The preparation of heterogeneous catalysts often includes challenging procedures to obtain a reproducible catalytic body. In many cases, the production procedure for a heterogeneous catalyst critically determines its selectivity and activity. While the preparation of catalysts was primarily done in a trial‐and‐error process up until the 1970s, it has evolved into a dynamic and economically important science that requires an interplay between many disciplines within chemistry and material sciences.^30^

**Figure 5:**
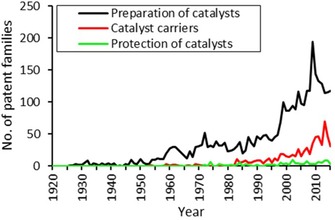
Development in the number of patent families within the field of catalyst fabrication in terms of catalyst carrier and the preparation and protection of catalysts.

Figure 5 reveals that within the domain of catalyst fabrication, the preparation of catalysts yields more patents than the related topics of catalyst carriers and protection of catalysts. Furthermore, patenting activities in the field of catalyst preparation show a significant increase starting in the mid 1990s, together with the overall increase in patenting activity already shown in Figure 3. As already pointed out above, the preparation of catalysts has been professionalized and connected to other research areas from the 1970s,^30^ eventually leading to a steep increase in patents in the 1990s.

**Figure 6:**
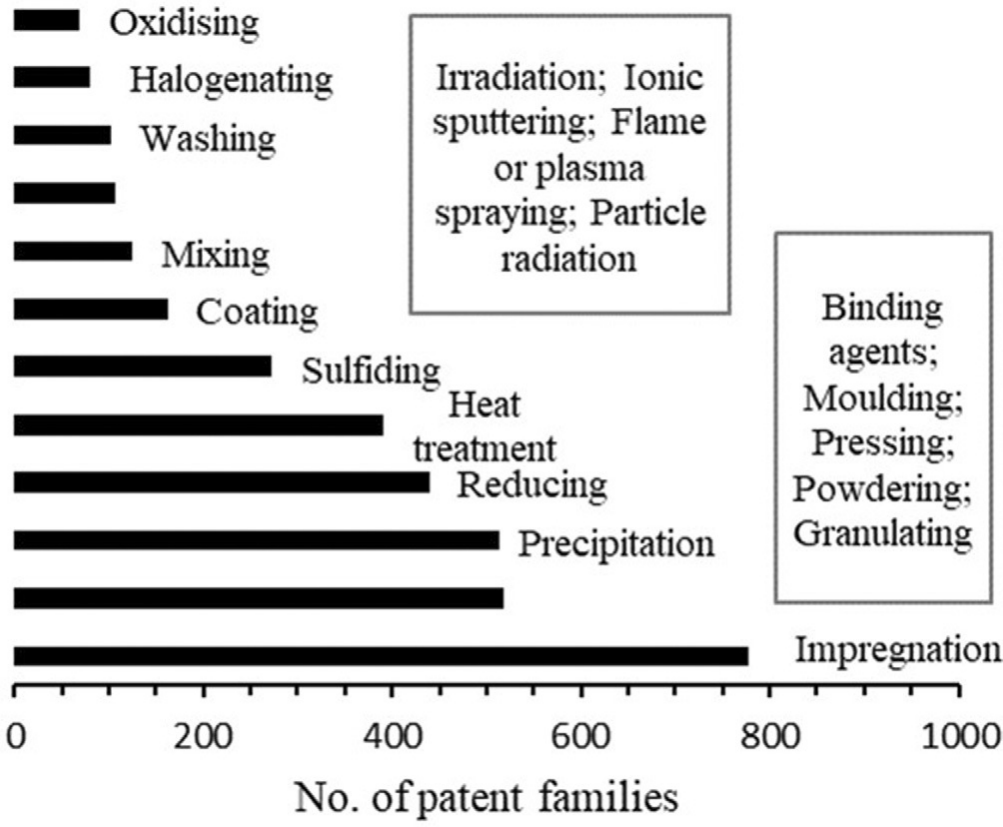
Overview of the number of patent families among the most important methods for the preparation of catalysts in hydrogenation from 1900–2015.

In order to better understand the driving forces of this increase, we conducted a more fine‐grained analysis of the preparation techniques for catalysts that is shown in Figure 6. The results reveal that impregnation as a preparation method (B01J37/0201) is a main driver of this increase. Impregnation describes a process in which a supported catalyst is prepared by exposing the catalyst support to a solution of the active metal component. The metal is then bound to the surface by ionic interactions or ligation of the metal atom.^31^ Examples include the impregnation of Ni on Al as the support to form a catalyst for the well‐known steam reforming process.^32^ The second most found patent classification is the mechanical treatment of solid catalyst bodies (B01J37/0009). These physical treatments involve processes such as molding, pressing, grinding, or granulating of the solids. This procedure influences mechanical stability, activity, and the regeneration procedure for catalyst reactivation. The third most common class in the subgroup analysis was the catalyst preparation by precipitation (B01J37/03). This preparation method can be used to fabricate a multicomponent catalytic body. Typically, a solution of the active metal component and the other components is treated with a precipitation agent, which is often an acid or a base. The precipitate is commonly collected by filtration, dried, shaped, and calcined to yield the active catalyst.^31^ A further subclass for the investigated hydrogenation catalysts is the reduction of the precatalyst (B01J37/16), which is often done by using molecular hydrogen. In that way, the catalytically active, reduced states of the metal components are achieved.^33^ The fifth most found subclass, heat treatment (B01J37/08), can be viewed in a general manner and describes all processes in which a catalyst is treated with heat during fabrication. The class “Sulfiding” (B01J37/20) was the sixth most found class in the subclass analysis. In the petrochemical industry, the raw natural gas‐oil contains considerable amounts of sulfur compounds that can poison catalysts of downstream processes and be detrimental to the performance of end‐product fuels.^34^ Therefore, the hydrodesulfurization (HDS) process is employed to remove sulfur from the crude oil by treatment with molecular hydrogen (i.e., by hydrogenation). To obtain highly active catalysts for the HDS process, the catalysts, mostly NiO or CoMo in combination with MoO_3_, are treated with sulfur‐containing compounds to form the respective active metal sulfides.^35^

#### 4.2.2 Protection of Catalysts

For some catalyst recipes, the protection of the catalyst (B01J33) is an important step in the fabrication process. For example, a thin Al_2_O_3_ layer between the catalytically active material and the underlying support has been patented for steam reforming reactions among others.^36^ The function of this layer includes the minimization of side reactions, the reduction of corrosion, and the reduction of thermal expansion stress.^36^ Interestingly, despite its economic relevance in large‐scare processes, we have observed only a small number of patents being filed in the domain of catalyst protection.

#### 4.2.3 Catalyst Carriers

Catalyst carriers, also known as supports, are substantial components of a catalyst system, which is why they are listed under their own patent classification (B01J32). It was observed that the patenting activity among catalyst carriers concerning the hydrogenation reaction was low over the time frame of investigation. Only from 2008 is a slight increase in patenting activity observed. A carrier of a catalyst can be any material that carries the active catalyst and it may have other functions than just structural fixation. Analogous to the field of catalyst protection, catalyst carrier materials are probably not explicitly classified.

### 4.3 Catalysts

**Figure 7:**
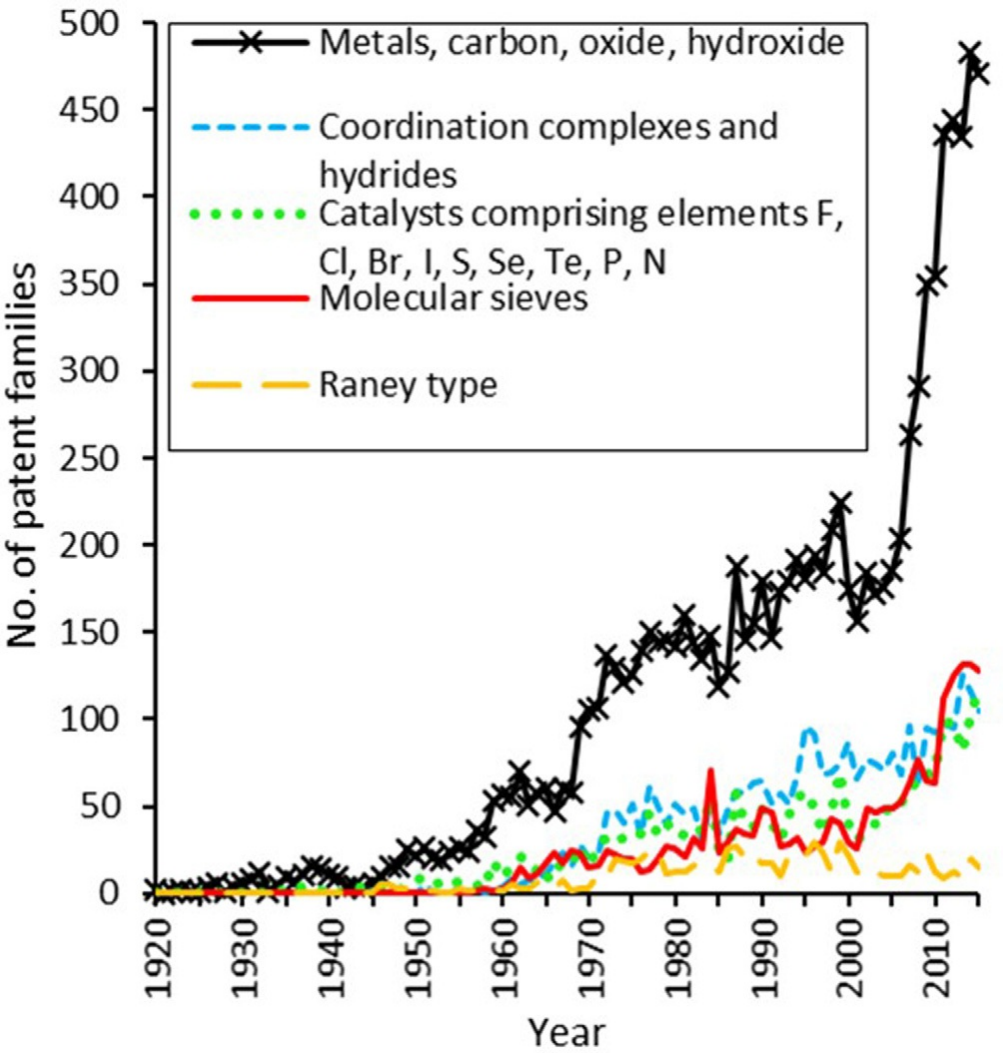
Development of the number of patent families across different types of catalysts.

Figure 7 shows the patent activity among different fields of catalyst types or materials from 1920–2015.

#### 4.3.1 Metals, Carbon, Oxides and Hydroxides

The highest number of patents are included in the IPC codes B01J21 and B01J23, which are analyzed together. These IPC classes include elemental forms of a variety of chemical entities, as well as the oxides and hydroxides of a variety of metals. Numerous typical heterogeneous hydrogenation catalysts such as PtO_2_ and Pd/C fall within these categories.

##### 4.3.1.1 Supporting Materials

IPC class B01J21 contains catalysts comprising the elements, oxides or hydroxides of Mg, B, Al, C, Si, Ti, Zr or Hf. While these elements are usually not the active components in hydrogenation catalysts, they are often used as supports for noble metals to produce an active hydrogenation catalyst or as promoters. The pure, catalytically active material – even in a highly porous form – would have a lot of precious material located at the inside and thus not available as a catalytic entity. For this reason, catalytically active material is spread out on a suitable supporting (inactive) material that can increase the contact area of the active sites with the reaction medium, which greatly improves the catalyst's cost efficiency. Promoters are ingredients that are not catalytically active but enhance a catalyst's performance, selectivity, or lifetime.^37^ The patenting activities for elements in the IPC class B01J21 are in decreasing order: Al, C, Si, combinations of Si and Al, and Ti. These are typical supporting materials for heterogeneous catalysts, since they are readily available materials.

##### 4.3.1.2 Active Metal Components

The IPC class B01J23 contains information concerning the active metal component of the catalyst. Table 1 shows an overview of the most patented active metal components in this IPC class based on their total number of patent families and their average annual growth rate within cumulated patent families for the period 2011–2015. All metals show positive growth rates which are, however, quite different in their magnitude. Patent family growth rates range from 1.3–9.4%, indicating almost stagnation for some metals *vs*. considerable growth rates of almost 10% for others. Table 1 summarizes our finding among the heterogeneous metal catalysts classified in IPC B01J23. In the table, the homogeneous catalyst metal section was derived from the more fine‐grained CPC codes (B01J2531 and respective subclasses thereof), which enable a detailed analysis of complexes with specific central atoms.

**Table 1:** Number of patent families and average annual growth rate in the cumulative number of patent families for for selected metals. Period 2011–2015.

**Heterogeneous catalyst metal**	**Patent families**	**Growth rate**
*Palladium*	1753	3.8%
*Nickel*	1106	7.1%
*Ruthenium, rhodium, osmium or iridium*	1023	4.8%
*Platinum*	837	4.2%
*Copper*	703	4.9%
*Cobalt*	426	6.6%
*Iron*	253	9.4%
**Homogeneous catalyst metal**	**Patent families**	**Growth rate**
*Rhodium*	457	1.8%
*Ruthenium*	352	3.4%
*Palladium*	223	1.8%
*Iridium*	223	2.8%
*Nickel*	178	2.0%
*Cobalt*	167	2.3%
*Platinum*	134	1.3%

The highest number of patent families (1753) was found for Pd. This is not surprising, since Pd is extensively used for hydrogenation reactions and is therefore of substantial economic and technological interest. Pd on C is an example of a hydrogenation catalyst used on industrial and laboratory scales.^38^, ^39^ The carbon support in these catalysts is advantageous, as it can be burned to recover the precious catalytically active Pd. A catalyst containing 1% Pd supported on Ca oxide/Al can be applied for the industrial hydrogenation of phenol.^40^ A medium growth rate of 3.8% was found for Pd in 2011–2015.

The second most patented metal among hydrogenation catalysts is Ni (1106 patent families, B01J23/755). In recent years, research has uncovered cross‐coupling reactions such as the Suzuki–Miyaura reaction that use Ni and boron reagents instead of the much more expensive Pd‐type reactions.^41^ A similar trend can be observed for the field of hydrogenation reactions, where research strives to harness more abundant elements, such as Ni, to replace rare metals.^42^ Another typical example is given by the hydrogenation of benzene described in Section 2. Here, a supported Ni oxide catalyst is applied for the hydrogenation of benzene to cyclohexane. Other significant applications of Ni catalysts are the hydrogenation of fatty acids and vegetable oils and fat hardening.^6^ The high average annual growth rate in the cumulated number of patent families of 7.1% compared to 3.8% for Pd, also shows the industrial importance and increasing interest in Ni as a heterogeneous catalyst and potential substitute for more expensive catalysts.^41^ The third largest group of patent families was Ru, Rh, Os, and Ir (1023 patent families, B01J23/46), while Pt (837 patent families, B01J23/42) is the fourth most abundant metal among the metals, oxides, and hydroxides. Pt oxide (also known as Adam's catalyst) is a common example of a Pt‐based hydrogenation catalyst. Originally developed to provide a reproducible catalyst system in the 1920s,^43^ Pt oxides are still used in supported catalysts in hydrogenation reactions as are a variety of Pt‐based catalysts for hydrogenation.^44^ The growth rate for the metals Ru, Rh, Os, and Ir amounts to 4.8% and for Pt to 4.2%. Consequently, compared to Pd, these metals show a faster increase in interest in the field of heterogeneous hydrogenation, albeit still less than Ni.

The majority of metals in this analysis are noble metals. Thus, the cost efficiency of a process partly depends on strategies to re‐use and recycle these catalytic bodies.

The next three most abundant metals among heterogeneous catalysts are Cu (703 patent families), Co (426), and Fe (253). Cu is often used within Cu chromite catalysts. These show a high selectivity towards C=O double bonds over C=C double bonds and are thus applied in the production of fatty alcohols from fatty acids.^45^ Co, for example, can be used in its Raney form as a catalyst to hydrogenate nitriles.^46^ Notably, recent developments in heterogeneous Co catalysis have emerged, describing a very active Co catalyst or the hydrogenation of C=O double and C≡N triple bonds.^47^ Only recently, a heterogeneous Fe‐catalyzed hydrogenation of nitroarenes was described, introducing a promising catalyst system on the basis of this abundant metal that operates under relatively mild conditions.^48^ All three metals are less expensive, which makes their use attractive in terms of prices for the raw catalyst metals. Especially for Co and Fe, this attractiveness can be shown by an increasing interest in both metals. Their growth rate for the period 2011–2015 amounts to 6.6% and 9.4%, respectively, which are next to Ni‐based heterogeneous catalysts the highest growth rates in this analysis. As we will see later, these growth rates are mainly driven by China.

In summary, the large number of patents filed in the domain of active metal components in the context of catalyzed hydrogenation reactions mirrors the well‐known practical importance of elemental metals and their oxides in the chemical industry. The amount of patents each year is growing across all metals considered. Therefore, heterogeneous catalysts – precious and non‐precious alike – will continue to play a major role in future chemical hydrogenation reactions.

#### 4.3.2 Coordination Complexes and Hydrides

The IPC class B01J31 describes coordination complexes and hydrides and thereby predominantly includes patents of homogeneous hydrogenation catalysts. The systems used for homogeneous hydrogenation hold a few intrinsic disadvantages that hamper their application in large‐scale chemical operations (e.g., often difficult separation from reaction mixture, difficult regeneration of catalysts). Their ability to effect selective transformations, tunability of complexes and the ability to hydrogenate substrates enantioselectively underline the importance of homogeneous hydrogenation catalysts for the fine chemical industry.^29^ The steady increase in patent activity since the 1960s indicates that active industrial research is still in progress to improve existing systems and develop new selective catalysts. For a more detailed analysis, we further investigate ligands and metals used in coordination complexes in the field of catalytic hydrogenation.

##### 4.3.2.1 Ligand Structures in Homogeneous Hydrogenation Catalysts

On a general level, the properties of homogeneous catalysts are determined by their two constituents: the central metal atom and its ligands. These ligands stabilize the complex (inhibiting degradation), tune the electronic properties (making the central atom more reactive), and create suitable surroundings about the reactive center by interactions with the substrate (enabling selective transformations). Thus, the reactivity of a complex depends not only on the character of the central atom, but also on its surrounding ligands. The defined chemical species used in homogeneous hydrogenation tend to have consequences for the patent activity on this field. Unlike for heterogeneous catalysts, a homogeneous catalyst's structure can easily be determined and the catalyst species can readily be synthesized by a competitor company in a short time. Hence, it seems reasonable to patent a catalyst structure for a certain application in order to prevent competitors from applying analogous processes.

**Figure 8:**
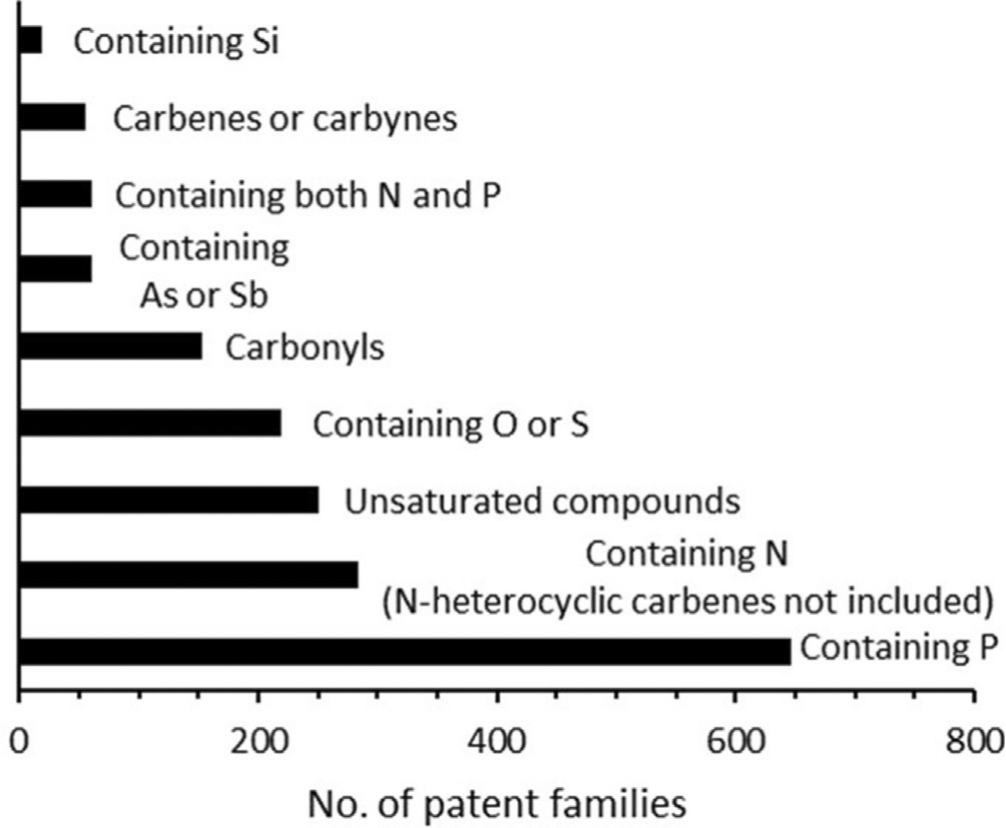
Overview of the number of patent families among the most important ligand structures for homogeneous hydrogenation catalysts from 1900–2015.

Since the ligand structure is of essential importance for the performance of homogeneous hydrogenation catalysts, a more detailed investigation of patent classifications among coordination complexes was pursued. The patent classifications among coordination complexes are organized by the ligand structure employed in the respective coordination complex. We adopted this classification (B01J2531 and subclasses) to produce the results shown in Figure 8.

Figure 8 shows that phosphorus‐containing ligands constitute the majority of complexes applied in patents. Today there are numerous ligand structures consisting of a phosphine moiety and they are widely applied in homogeneous hydrogenation reactions as modular and tunable entities for reaching the desired reactivity in catalytic systems.^49^, ^50^, ^51^ An example is given by the homogeneous enantioselective hydrogenation to produce l‐DOPA, as shown above in Scheme 3.

Nitrogen‐containing ligands are the second most frequently protected class. In this ligand class, chiral diamines play a central role such as in the hydrogenation catalysts developed by Noyori (for which he was awarded the Nobel prize in 2001). Today, these catalysts are state‐of‐the‐art for the enantioselective hydrogenation of C=O double bonds.^52^, ^53^ An impressive example of the system's utility can be found in the enantioselective synthesis of the antidepressant drug fluoxetine reported by Noyori using chiral diamines as ligands in addition to phosphorus‐containing ligands.^54^

Unsaturated compounds (cyclopentadienyls, olefins) are the third most found group of ligands. Although they are sometimes easily replaceable, unsaturated compounds may substantially tune the reaction properties of the complex. As an example for this group, cyclopentadienyl fragments are readily found in transfer‐hydrogenation catalysts.^55^ These are commonly viewed to be attached to the metal throughout the catalytic process and modify the activity and selectivity of a homogeneous catalyst species. Fourth and fifth are catalyst species including ligands that contain O or S, and carbonyls. Other ligand species play a minor role in homogeneous hydrogenation catalysis.

##### 4.3.2.2 Metal Components in Homogeneous Hydrogenation Catalysts

For a detailed analysis of metals used as a central atom in coordination complexes (homogeneous catalysts), we further investigated the CPC class B01J 2531, which gives additional information about the central metal atom of the coordination complex. Table 1 shows an overview of the most patented metals in this class, based on their total number of patent families, and their average annual growth rate in cumulated patent families for the period 2011–2015. We find that Rh (457 patent families), Ru (352), Pd (223) and Ir (223) are the most frequently patented metals among homogeneous hydrogenation catalysts. As exemplified by Wilkinson's catalyst, which is an early example of a very active Rh complex for homogeneous hydrogenation and one of the most studied, the field of homogeneous catalyst metal components was consequently found to be dominated by this metal.^56^, ^57^, ^58^ Ru is found as the active metal in Noyori‐type catalysts mentioned in the previous section and is also found in great abundance in the patent literature.^52^, ^53^ Furthermore, Ir‐catalyzed homogeneous hydrogenation reactions are also well known in the literature, including enantioselective methods.^59^ When looking at the average annual growth rate in the cumulated number of patent families, it becomes evident that Ru shows the highest growth rate (3.4%) among the metals analyzed. Pt shows the lowest growth rate in recent years with 1.3%. When comparing the annual patent growth rates of heterogeneous and homogeneous hydrogenation catalysts in Table 1, we find that heterogeneous catalysts grow at a considerably higher rate than their homogeneous counterparts, which is in alignment with the industrial applicability of the reaction types. Although the use of base metals would also be highly desirable in homogenous catalysis, and technologies are emerging in this field,^60^ the shift towards the use of base metals that was observed in heterogeneous catalysis was not found in the patent landscape of homogeneous catalysis.

#### 4.3.3 Economic and Regional Investigation of Metals in Hydrogenation Catalysts

While the overall technological interest among the analyzed metals in heterogeneous and homogeneous hydrogenation was shown in Sections 4.3.1 and 4.3.2, we further analyze the economic quality and regional aspects of those metals. A well‐established patent indicator for the economic quality is the share of triadic patents of a technology.^61^ Triadic patents are patents filed in patent offices in the USA (USPTO), in Europe (EPO), and in Japan (JPO). Because these regions are economically strong markets, a high share of triadic patents for a specific technology indicates a high economic value for this technology.^61^ Thus, we next investigated triadic patents for all metals analyzed in Sections 4.3.1 and 4.3.2. Furthermore, we give an overview of regional differences among these metals for heterogeneous and homogeneous catalysts in hydrogenation.

When choosing a metal catalyst for hydrogenation, a variety of factors have to be taken into account in order to realize an economically reasonable process:

Catalyst production, i.e., the price of the raw metal and other catalyst components, the preparation process.
Catalyst activity and selectivity, i.e., the ability of the given catalyst to mediate the desired chemical reaction selectively.
Catalyst regeneration, i.e., reusability and processes to reactivate the catalytic body.
Catalyst reproducibility, i.e., the reliability of a given catalyst to give satisfactory outcome of the applied process.

Choosing a catalyst and especially the central active metal component has to take into account all these factors and often requires managing trade‐offs between conflicting dimensions.

For the IPC code B01J23, which was earlier determined to predominantly include heterogeneous catalysts, the majority of catalytic bodies are classified to contain Pd, Ni, Ru, Rh, Os, Ir, Pt, Cu, Co and Fe. This is not surprising since these metals are commonly found in heterogeneous hydrogenation catalysts.

Among these metals, the difference in the share of triadic patents, shown in Table 2, is noticeable. In our analysis, the highest share of registered triadic patents amounts to 38% for Ru, Rh, Os, Ir, indicating their high economic value in industrial applications. As an example from the field of homogeneous hydrogenations, Rh is often used as an active catalyst to hydrogenate aromatics,^62^ while heterogeneous Ru catalysts can be used for the selective hydrogenation of C=O double bonds over C=C double bonds.^63^ An average share of triadic patent families among the analyzed metals was found for Pd (33%), Pt (35%), Cu (31%), Co (35%) and Fe (30%), while the share of triadic patent families for Ni was found to be significantly lower (26%). Especially in the case of Fe and Ni, a detailed patent analysis reveals that the main reason for these differences is the substantial increase in patenting activities in China since the 1990s. Due to the strong increase in the number of patents filed in China there is a significant increase in the number of patents in these fields overall, indicating the increasing importance of this region and market, eventually reducing the relative importance of the other regions.

**Table 2:** Share of registered triadic patent families for metals in heterogeneous and homogeneous hydrogenation for the period 2011–2015.

**Heterogeneous catalysts**	**Triadic patents**
*Ruthenium, rhodium, osmium or iridium*	38%
*Platinum*	35%
*Cobalt*	35%
*Palladium*	33%
*Copper*	31%
*Iron*	30%
*Nickel*	26%
**Homogeneous catalysts**	**Triadic patents**
*Ruthenium*	50%
*Iridium*	48%
*Palladium*	47%
*Platinum*	43%
*Rhodium*	38%
*Nickel*	38%
*Cobalt*	29%

**Figure 9:**
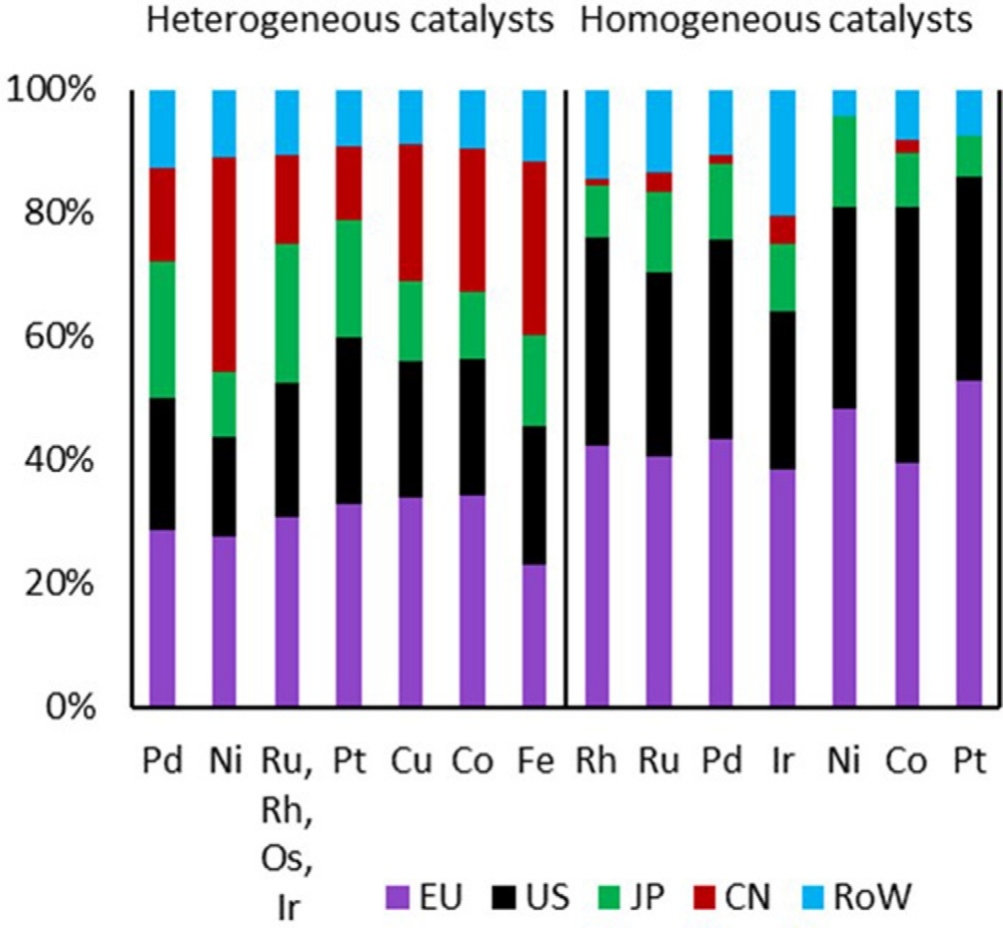
Geographical distribution of patent families classified by priority country for heterogeneous and homogeneous hydrogenation catalysts from 1900–2015.

Furthermore, the increasing relevance of China as a market and innovator in the field of heterogeneous Ni and Fe catalysts is also displayed by the share of patent families registered in China as a priority country compared to the other regions (see Figure 9). Concerning Ni, the highest number of patent families was registered in China as a priority country (34% of all patent families), followed by Europe [please note: for “Europe”, patent families registered in following patent offices as priority country were counted: EPO, Germany, France, and Great Britain] (28%), the USA (16%), and Japan (11%). A similar constellation was observed for Fe, where 28% of all patent families are registered in China as a priority country, followed by Europe (23%), the USA (23%), and Japan (15%). Specifically, Figure 9 shows that patents in the field of heterogeneous catalysis that are first filed in China focus on the cheaper metals Ni, Fe, Co and Cu, while the overall patent activity for the expensive platinum group metals is considerably lower.

For the IPC B01J2531, which describes homogeneous catalysts, the majority of catalytic bodies are classified to contain Rh, Ru, Pd, Ir, Ni, Co, and Pt. When comparing these metals with metals used in heterogeneous catalysis, it becomes evident that in general the share of triadic patent families is considerably higher for homogeneous catalysts. The high share of triadic patents among this subclass may reflect efforts to protect the technologies classified here. As mentioned previously, the structure of a homogeneous complex can often be identified and reproduced much more easily than the structure of a heterogeneous catalyst, and thus might be protected more restrictively. The highest shares of triadic patent families among the homogeneous catalysts were registered for Ru (50%), Ir (48%), and Pd (47%) (see Table 2). Thereafter Pt (43%), Rh, and Ni (both 38%) follow, while the lowest share of triadic patent families can be observed for Co (29%).

When analyzing the geographical distribution of patent families among priority countries, the minor role of China among patent applications in homogeneous catalysts can be seen for the period from 1900–2015 (see Figure 9); on average the patent families registered in all analyzed metals amount just to 2%. The largest patenting activity is found in Europe and the USA. On average, both regions contribute together with 76% of patent families registered in this field.

#### 4.3.4 Raney‐Type Catalysts

As seen in Figure 7 the patent activity for Raney‐type catalysts remains on a constantly low level throughout the whole time frame investigated. Raney‐type catalysts, the most prominent example being Raney nickel, were originally developed to provide a reproducible catalyst for hydrogenation reactions. Raney nickel is usually manufactured by melting Ni and Al to provide an alloy. The alloy is crushed into fine particles. Subsequently, the Al is extracted by gradual addition of caustic soda solution, leaving a highly porous Ni catalyst that is obtained after washing and activation. It is still used as a highly active laboratory and small‐scale hydrogenation catalyst, however, its high production costs compared to other hydrogenation catalysts hamper its general application in industrial hydrogenation nowadays. Nevertheless, there are still processes that employ these catalyst types.^6^

#### 4.3.5 Catalysts Comprising Elements F, Cl, Br, I, S, Se, Te, P, N

The class B01J27 describes catalysts that contain one of the following elements: F, Cl, Br, I, S, Se, Te, P, N. Most often, these elements are added either for structural reasons or as promoters. Since there may be many reasons for adding one of these elements, a structured search of the subclasses was considered not to deliver further clear information. As can be seen in Figure 7 the patent activity in this class is reasonably comparable to that for the following B01J29 class, showing increasing patenting from around year 2010. It is worth noticing that the patents exemplarily investigated in this class were of heterogeneous nature.

#### 4.3.6 Molecular Sieves

The patent activity for hydrogenation catalysts on the basis of molecular sieves has experienced a peak in 1984 and a steady increase since the year 2000 (see Figure 7). Molecular sieves (B01J29), including zeolites, are crystalline porous aluminosilicate materials with small pore diameters of typically <50 nm. Especially zeolites can react as Lewis acids because of their high Al content. With respect to hydrogenation reactions, the porous materials can be impregnated with metal hydrogenation catalysts, creating a bifunctional catalyst that can not only react to add hydrogen to unsaturated parts of a molecule but also induce acid‐promoted reactions.^64^

These types of catalysts are especially useful for oil refinery processes involving catalytic hydrocracking.^65^ In these processes, low‐value heavy gas oils derived from natural oil can be converted into light gases, gasoline, jet fuel or diesel oil, depending on the reaction conditions. At temperatures of 350–450 °C and a hydrogen pressure of 60–200 atm, the reaction can take place over the zeolite catalysts.^6^ These catalytic systems were introduced in 1964 by the Union Oil Company, as they allowed conversion of a wider range of high boiling‐point starting materials to more valuable and shorter hydrocarbons.^66^

A detailed analysis of the IPC and CPC codes revealed that the five most abundant classifications all describe crystalline aluminosilicate zeolites. This is expected as these comprise the typical zeolitic catalyst for hydrocracking. The significant increase in patent activity since 2000 indicates that molecular sieve catalysts are still widely used and are continuously being improved to develop optimal hydrocracking catalysts for the selective generation of high‐value fuels. For the petrochemical industry, slight increases in catalyst activity or selectivity can substantially increase a process's profitability because of the immense scale. Development of suitable catalysts can therefore have a significant impact on the economics of the overall process.

### 4.4 Catalyst Investigation and Further Manipulation

#### 4.4.1 Physical Properties

**Figure 10:**
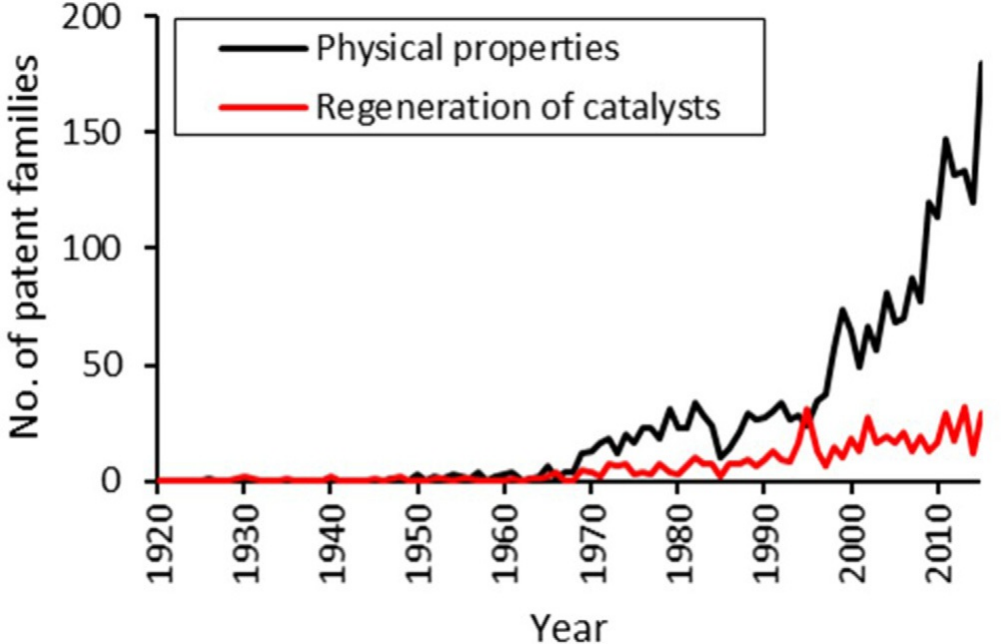
Number of patent families protecting the physical properties of hydrogenation catalysts and their regeneration.

The B01J35 class distinguishes the physical properties of catalytic bodies that have been determined by appropriate analytical techniques. Since all the properties described in this patent class only apply to solids, we conclude that these are heterogeneous catalysts. Figure 10 shows that in the 1970s, the characterization of catalysts started to follow a more strictly scientific approach.^30^ This could imply that more and more effort was devoted to the analysis and the understanding of structure‐activity relationships in heterogeneous catalysts, thus moving away from a “black‐box” perception of heterogeneous catalysts.^67^

**Figure 11:**
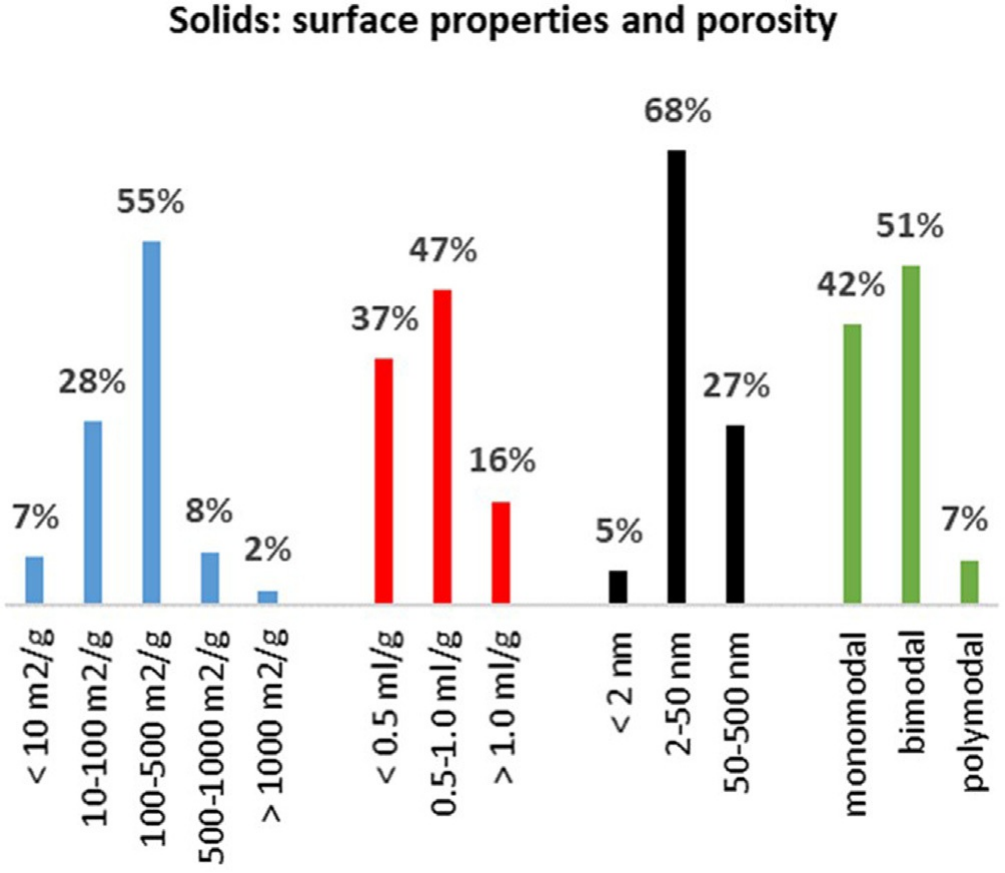
Overview of the distributions of patent families in different research fields among catalysts surface properties and porosity from 1900–2015. [Blue: surface area; orange: pore volume (ml g^−1^); grey: pore volume (nm); green: pore distribution].

Based on the more detailed CPC codes, the B01J35 class allows a detailed view of the properties of patented catalysts in heterogeneous hydrogenation. It can be assumed that the distribution of the properties, as seen in the patent data, can give a picture of the actual properties of catalysts applied in chemical processes. Figure 11 shows that certain properties occur more often than others. The properties are specific surface area, average pore volume and average pore diameter, and they are important for the catalyst's selectivity, activity, and recyclability. Moreover, these geometric properties are related and therefore interdependent on each other.

The specific surface area of catalysts of 100–500 m^2^ g^−1^ is the most common category found in the evaluated patents (55% of the patents classified in this section). In principle, the catalyst specific surface should correlate with the speed of the catalyzed reaction as the reaction mixture is in contact with a higher fraction of the catalytic body per unit. However, the specific surface area cannot be extended without influencing other geometric parameters. It seems that the surface area of catalysts of 100–500 m^2^ g^−1^ represents an optimum, when considering the other parameters.

In heterogeneous catalysis, the pore volumes substantially influence the kinetics of the catalyzed reaction. Here, researchers face a trade‐off between favorable catalyst properties when deciding which pore size to use. On the one hand, a large pore size is desirable because it enables sufficient mass transport inside the catalyst, so that the catalytic sites can be provided with enough starting material and the products are released into the solution at a sufficient speed. Furthermore, large pores prevent the channels in the catalyst from blocking and eventual deactivation. On the other hand, having large pores stands in conflict with providing a high number of catalytic sites per unit volume (i.e., a high surface area) and makes the catalyst less resistant to physical impact due to its high porosity. The pore diameter of a catalytically active body can also be used to introduce selectivity. For example, a catalyst with small pores allows small molecules to enter, while excluding larger ones.^68^, ^69^

For the classification of pore sizes, different scales have been developed. The two most commonly used classifications for pore volumes are 0.5–1.0 mL g^−1^ (47%) and <0.5 mL g^−1^ (37%).

According to IUPAC, pore sizes are grouped into three categories based on their diameter: micropores (<2 nm), mesopores (2–50 nm) and macropores (> 50 nm).^70^ We adopted this classification for our analyses. With regard to the hydrogenation patents classified, the majority of patents fall within the category of mesopores (68%), followed by macropores (27%), and micropores (5%).

#### 4.4.2 Regeneration of Catalysts

IPC class B01J38 describes processes for catalyst reactivation and regeneration. During the catalytic process, catalysts may lose their activity due to various reasons. Major causes for the loss of activity include poisoning, fouling, thermal degradation, mechanical damage, and corrosion/leaching. These processes are further described in Table 3.^71^

**Table 3:** Five main reasons for the loss of catalytic activity.^62^

	**Cause of activity loss**
*Poisoning*	Strong adsorption of impurities in the reaction mixture *chemically* blocking the active sites
*Fouling*	Solid deposition on the catalysts surface *physically* blocking the active sites
*Thermal degradation*	Sintering, evaporation or diffusion into an inert support of catalytically active chemical entities reducing the amount of catalytically active surface
*Mechanical damage*	Disintegration of catalytically active entities by mechanical stress, destruction of structure and loss of catalytically active sites
*Corrosion/Leaching*	Dissolution of active sites or the structure of a catalyst support, loss of active sites and structure

Catalysts often contain highly precious elements like noble metals or may be difficult to prepare. This makes catalyst regeneration an economic imperative and it is therefore desirable to maximize the lifetime of a given catalytic body. Despite the economic relevance of catalyst regeneration, patenting activities in this domain are relatively low, as shown in Figure 11. Only from around 1990 was a slight increase in patenting activity observed. A possible explanation is that catalyst preparation and process control are considered to be the major sources of competitive advantage, while catalyst regeneration is not as critical and therefore less patented.

We believe that this abundancy is correlated with the fact that catalyst preparation and operation is a much more valuable process knowledge to be protected by the respective company. A process for catalyst regeneration could then be derived after catalyst development and be kept confidential without warranting the resources for the filing of a related patent.

### 4.5 Results of Keyword‐Based Search

**Figure 12:**
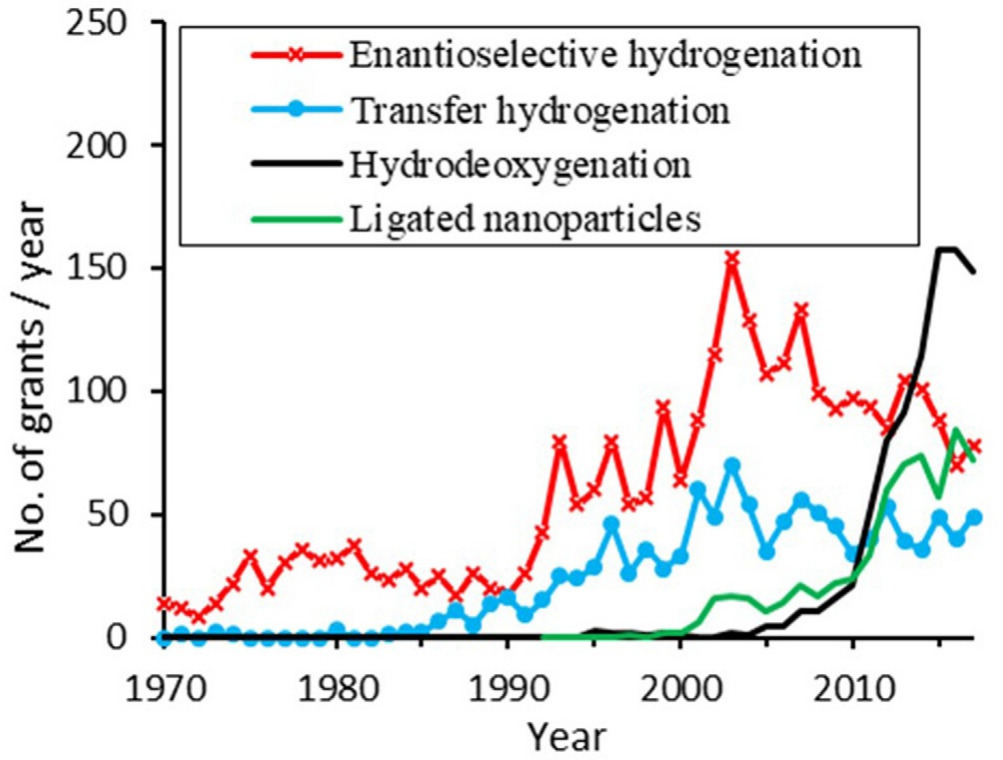
Results of keyword‐based search for selected hydrogenation reaction types.

In addition to using the predefined IPC and CPC classifications, we complement our analysis with a keyword‐based approach. In so doing, developments in hydrogenation catalysis that are not captured by the above classifications are illuminated. Figure 12 shows the patent grants for enantioselective hydrogenation, transfer hydrogenation, hydrodeoxygenation, and the use of nanoparticles from 1970–2017. As this figure shows, the economic interest in enantioselective hydrogenation, after peaking two years after the Nobel prize was rewarded to Noyori in 2001, continues to be at a substantial level. Innovations in transfer hydrogenation are being continuously patented at a rate of around 50 grants per year. Furthermore, nanoparticles are finding increasing use for catalyzed hydrogenation reactions. Patent grants in this relatively young field have seen a significant increase since around the year 2010, indicating its emerging industrial relevance. Perhaps most noteworthy, the current number of patent grants in field of hydrodeoxygenation (HDO) surpasses all others. HDO describes a series of reactions that aim at converting biomass‐derived substrates into renewable fuels and chemicals.^72^ The urge to fight climate change accelerates the development of this technology at industrially relevant scales. An analysis of metal‐organic frameworks (MOFs) was also done, but industrial applications are still in their infancy, research is mainly driven by universities, and therefore overall patents are scarce. In terms of the technology life cycle, MOFs are an emerging technology.

## 5 Conclusions

This review presents an overview of the patent landscape of hydrogenation catalysts.

Among the different catalyst types, active metal components and catalyst support have the highest competitive impact, but molecular sieve‐based catalysts are characterized by an increasing competitive impact in recent years, exceeding catalysts based on coordination complexes and hydrides. On breaking down patent activity of different ligand structures of homogeneous hydrogenation catalysts, phosphorus‐containing ligands constitute the majority of applied complexes, followed by ligands containing nitrogen and unsaturated compounds. Within the group of heterogeneous hydrogenation catalysts, palladium is found to be the most prevalent metal component, while rhodium is most frequently used among homogeneous hydrogenation catalysts. However, the patent analysis revealed for both heterogeneous and homogeneous hydrogenation that other metal components are showing a higher patent activity in recent years, mainly driven by patenting activities in China. A geographical analysis revealed that China has a relatively strong focus on heterogeneous catalysts and abundant metals like nickel or iron. Furthermore, an increasing R&D effort is devoted to the physical properties of solids.

While the classification‐based search was useful to differentiate between different technologies in hydrogenation catalysis, complementing the results with an informed keyword‐based approach is worthwhile. Hydrodeoxygenation stood out in this analysis, showing a rapidly increasing body of patents granted per year.

According to the technological life cycle concept, hydrogenation catalysis can be considered to be in its growth stage, as indicated by high market penetration and simultaneously accelerating patenting activities. The number of patent families in heterogeneous hydrogenation grows twice as fast as the number of patent families in homogeneous hydrogenation, which is in accordance with its industrial applicability. Finally, catalysis whose purpose aligns with contemporary societal challenges, such as climate change, will feature above average growth rates in patents.

## Biographical Information

*Marius Stoffels* received a PhD from the Westfälische Wilhelms Universität Münster under the supervision of Prof. Jens Leker. His research is positioned at the nexus of science and business. Current research includes the impact of digital technologies on value creation in the chemical and pharmaceutical industry.

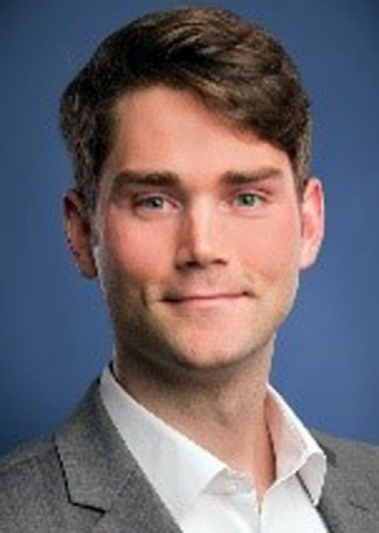

## Biographical Information

*Thomas Hamadi* is Innovation and Patent Manager at Infineon Technologies AG and obtained his PhD from the Institute of Business Administration at the Department of Chemistry and Pharmacy, University of Münster. His research focus is on patent analysis and innovation management in high‐tech industries.

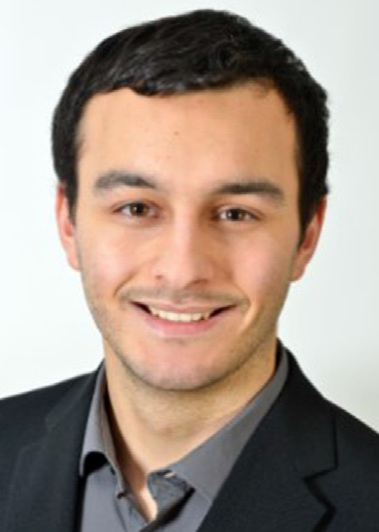

## Biographical Information

*Jens Leker* is a Full Professor of Business Administration in the Natural Sciences at Westfälische Wilhelms‐Universität Münster. He is Editor‐in‐Chief of the *Journal of Business Chemistry* and his research includes corporate strategy, patent analysis, technology foresight, and innovation management in research‐intensive industries.

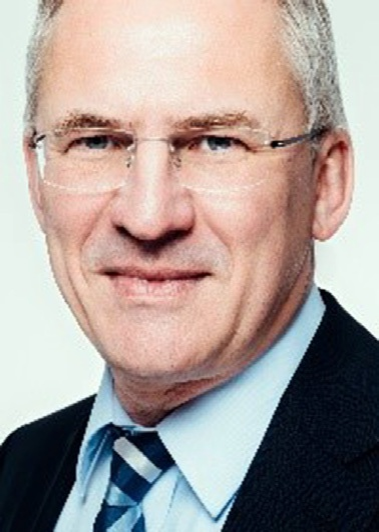

## Biographical Information

*Felix Klauck* studied chemistry at the University of Cologne and the RWTH Aachen University. In his Master studies he joined the group of Prof. J. C. Anderson at the University College London for a research stay. He pursued his doctoral studies with Prof. F. Glorius at the University of Münster working on metal‐catalyzed C−H activation and photoredox catalysis. After obtaining his doctoral degree in the year 2019 he started working at INEOS Styrolution as a Research Specialist for styrene‐based polymers.

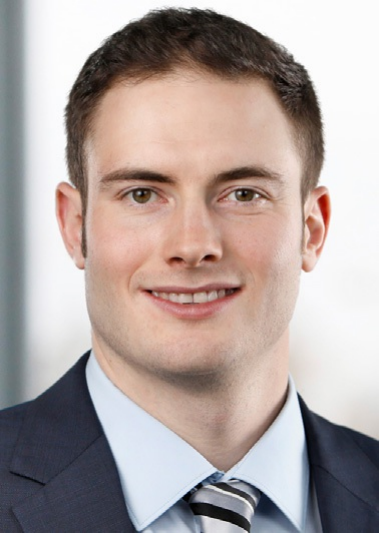

## Biographical Information

*Frank Glorius* is a Full Professor of Organic Chemistry at the Westfälische Wilhelms‐Universität Münster. His research program focusses on the development of new concepts for diverse areas of catalysis such as photocatalysis, C−H activation, smart screening and data‐based technologies, N‐heterocyclic carbenes (NHCs) in organocatalysis and as surface modifiers and in (asymmetric) arene hydrogenation.